# Characterization of *SLC22A18* as a tumor suppressor and novel biomarker in colorectal cancer

**DOI:** 10.18632/oncotarget.4681

**Published:** 2015-06-28

**Authors:** Yeonjoo Jung, Yukyung Jun, Hee-Young Lee, Suyeon Kim, Yeonhwa Jung, Juhee Keum, Yeo Song Lee, Yong Beom Cho, Sanghyuk Lee, Jaesang Kim

**Affiliations:** ^1^ Ewha Research Center for Systems Biology, Seoul, Korea; ^2^ Department of Life Science, Ewha Womans University, Seoul, Korea; ^3^ Samsung Biomedical Research Institute, Seoul, Korea; ^4^ Department of Surgery, Samsung Medical Center, Sungkyunkwan University School of Medicine, Seoul, Korea

**Keywords:** SLC22A18, tumor suppressor, colorectal cancer, G2/M arrest, KRAS

## Abstract

*SLC22A18*, solute carrier family 22, member 18, has been proposed to function as a tumor suppressor based on its chromosomal location at 11p15.5, mutations and aberrant splicing in several types of cancer and down-regulation in glioblastoma. In this study, we sought to demonstrate the significance of *SLC22A18* as a tumor suppressor in colorectal cancer (CRC) and provide mechanistic bases for its function. We first showed that the expression of *SLC22A18* is significantly down-regulated in tumor tissues using matched normal-tumor samples from CRC patients. This finding was also supported by publically accessible data from The Cancer Genome Atlas (TCGA). Functionally, SLC22A18 inhibits colony formation and induces of G2/M arrest consistent with being a tumor suppressor. Interestingly, suppression of *KRAS* by RNA interference promotes *SLC22A18* expression, and expression of *SLC22A18* in turn inhibits KRAS^G12D^-mediated anchorage independent growth of NIH3T3 cells indicating a mutual negative interaction. Finally, we evaluated diagnostic and prognostic values of *SLC22A18* using clinical and gene expression data from TCGA which revealed a significantly worse long-term prognosis for patients with low level *SLC22A18* expression. In sum, we established *SLC22A18* as a tumor suppressor in colon epithelial cells and propose that *SLC22A18* is potentially a marker of diagnostic and prognostic values.

## INTRODUCTION

CRC is currently the one of the highest-ranked cancers in terms of both mortality and incidence rates [1]. In the United States alone, nearly 140,000 new cases and over 50,000 deaths associated with CRC are projected for 2014 [2]. Although such numbers signify a slight decline of colorectal cancer cases in United States, the incidence has been on the rise in other parts of the world including many populous Asian countries where colonoscopic screening for early detection is still limited [3, 4].

Clearly, novel molecular markers would be beneficial both for better diagnosis and prognosis and for characterizing the molecular mechanism of CRC development. We have previously reported isolation of novel markers of CRC from meta-analysis of publicly available gene expression data [5]. Of the 34 genes we have isolated, we validated 9 of them to be significantly up-regulated in cancer tissues compared to normal surrounding colon tissues. At least some of these genes (*ECT2, ETV4, DDX21, RAN, S100A111, RPS4X, HSPD1, CKS2* and *C9orf140*), three of which have been shown to be activated by c-MYC or KRAS, likely contribute to oncogenic processes [5].

In contrast, the genes down-regulated in cancer tissues represent potential tumor suppressors. Here, we report characterization of *SLC22A18*, a member of a large family of cytoplasmic membrane associated transporters [6], as a potential tumor suppressor of CRC. Also known as *IMPT1, BWR1A, TSSC5* and *ORCTL2*, *SLC22A18* is located in 11p15.5, a chromosomal region which is frequently deleted in a variety of cancers [7] and encompasses several imprinted genes most of which including *SLC22A18* show preferential expression of the maternal allele [8, 9]. Taken together with that the gene silencing and the loss-of-heterozygosity (LOH) involve mostly the maternal allele [10–12], it has been proposed that at least one tumor suppressor gene is present in this chromosomal region. Missense mutations of *SLC22A18* have been found in a variety of cancer types including breast cancer, lung cancer and rhabdomyosarcoma, and aberrant splicing of this gene has been reported in multiple Wilms' tumor cases [13, 14]. Together, these reports suggest that *SLC22A18* functions as a tumor suppressor in certain cell types. Consistently, Chu and coworkers showed that down-regulation of *SLC22A18* was frequently associated with glioma development and that ectopic expression of SLC22A18 led to reduced proliferation of U251 glioma cells *in vitro* and *in vivo* [15]. Furthermore, low *SLC22A18* expression has been reported to be correlated with poor prognosis for glioma patients and breast cancer patients [16, 17].

Here, we provide for the first time evidences indicating that SLC22A18 functions as a tumor suppressor in CRC. The gene is down-regulated in CRC and can retard the growth of CRC cell lines by inducing G2/M arrest. Remarkably, expression of SLC22A18 is negatively regulated by KRAS, and SLC22A18 in turn appears to negatively regulate KRAS signaling. In addition, our preliminary analysis of public data from TCGA indicates that *SLC22A18* may also be a potential prognostic marker of CRC.

## RESULTS

### Down-regulation of *SLC22A18* in CRC tissues

We have previously reported isolation of 34 genes differentially expressed in CRC from public data analysis and confirmation of 9 among them as potential biomarkers of CRC based on quantitative RT-PCR results using matched normal and tumor tissues [5]. These had been selected based on significantly elevated expression in tumor tissues of 10 randomly selected patients (*P*-value < 0.01; Supplementary Figure 1). Several genes including *SLC22A18* also showed significantly differential expression but with lower levels in tumor tissues (Supplementary Figure 1). *SLC22A18*, a member of membrane bound solute carrier gene super family, has been proposed to be a tumor suppressor based on its chromosomal location in 11p15.5 as well as frequent mutations found in various tumors and was thus selected for further analyses.

**Figure 1:**
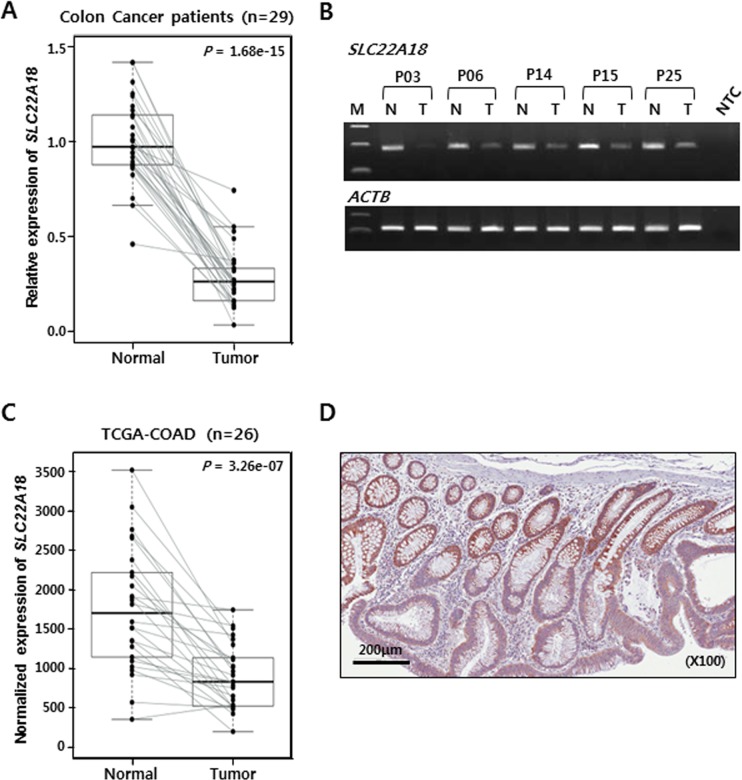
Decreased expression of *SLC22A18* in CRC tumors **A.** Box-plot of quantitative real-time RT-PCR results. Case-matched normal and tumor samples from CRC patients are represented as 29 independently connected lines. The normalized expression value with *ACTB* and *HPRT* of 1 is arbitrarily set so as to encompass all levels of expression. Each point is the average of quantitative analyses in duplicate reactions. The bold line in the middle point of each box indicates the median level. A significant (*P*-value, 1.68e-15) decrease in the expression of *SLC22A18* was noticed for tumor tissues compared to normal tissues from surrounding area. **B.** Conventional RT-PCR analysis to confirm results from the real-time RT-PCR. Normal (N) and tumor (T) tissues from 5 representative CRC patients were examined. Tumor tissues show decreased *SLC22A18* expression. *ACTB* is amplified as the control. NTC stands for no template control. **C.** Examination of *SLC22A18* expression from the TCGA COAD data set. Only the RNA-Seq data with both tumor and matched normal tissues were used. Analysis of 26 colorectal cancer cases confirmed that *SLC22A18* expression is decreased in tumor samples in 25 out of 26 cases (*P*-value, 3.26e-7) in excellent agreement with our patient cohort. **D.** Immunohistochemical image of CRC sample containing both normal and tumor tissues stained with anti-SLC22A18 antibody. Normal intestinal cells in the top half of the tissue show stronger staining than cancer cells in the bottom half of the tissue.

First, real-time RT-PCR assay was carried out with samples from 29 patients. Without exception, the expression was down-regulated in tumor tissues compared to matched normal tissues (Figure 1A). This was also confirmed by conventional RT-PCR followed by gel electrophoresis using 5 representative patient samples (Figure 1B). We were able to obtain RNA-seq data from 26 normal-tumor matched samples of colon adenocarcinoma (COAD) from TCGA and determine normalized expression values of *SLC22A18*. Consistent with RT-PCR results from our patient samples, virtually all tumor tissues showed lower expression of *SLC22A18* than matched normal tissues (Figure 1C). We obtained paraffin-embedded tumor tissues from an independent group of patients and examined SLC22A18 expression by immunohistochemical staining. Consistent with results from RT-PCR, tumor areas from all samples examined showed down-regulated expression of SLC22A18 (Figure 1D and Supplementary Figure 2).

We next investigated the mutation status of *SLC22A18*. First, using our patient genomic DNA samples from normal and tumor tissues, we PCR-amplified all coding portions of *SLC22A18* and examined the nucleotide sequence by Sanger sequencing. We found no mutations aside from novel polymorphisms and no evidence for LOH (data not shown). All cases of heterozygosity found in tumor tissues were also seen in normal tissues and vice versa. Second, we examined exome sequences of 151 colon adenocarcinoma patients with matched normal-tumor samples from TCGA database. Our analysis using GATK (version 2.5.2) program to call for heterozygous loci and VarScan2 (version 2.3.5) program to call for LOH revealed only two cases of LOH out of 151 patients (data not shown). These data together indicate that by and large mutations or LOH is not the cause for lowered expression of *SLC22A18* in colorectal cancer cases.

We have expanded the analysis of TCGA RNA-seq data to all cancer types with matched normal-tumor samples. Down-regulation in tumor tissues appears to be restricted to COAD as other types of cancer showed no significant down-regulation in tumor tissues (Supplementary Figure 3). Although rectal cancer showed down-regulation in 6 out of 6 tumor tissues, the statistical significance was not attained due to the limited number of patient samples. Still, if the COAD and rectal cancers are pooled together into CRC, the correlation should be even more highly significant. Of note, for lung adenocarcinoma, thyroid cancer and breast cancer, *SLC22A18* level appears to be higher in tumor tissues (Supplementary Figure 3).

### Inhibition of colony formation by SLC22A18

**Figure 2:**
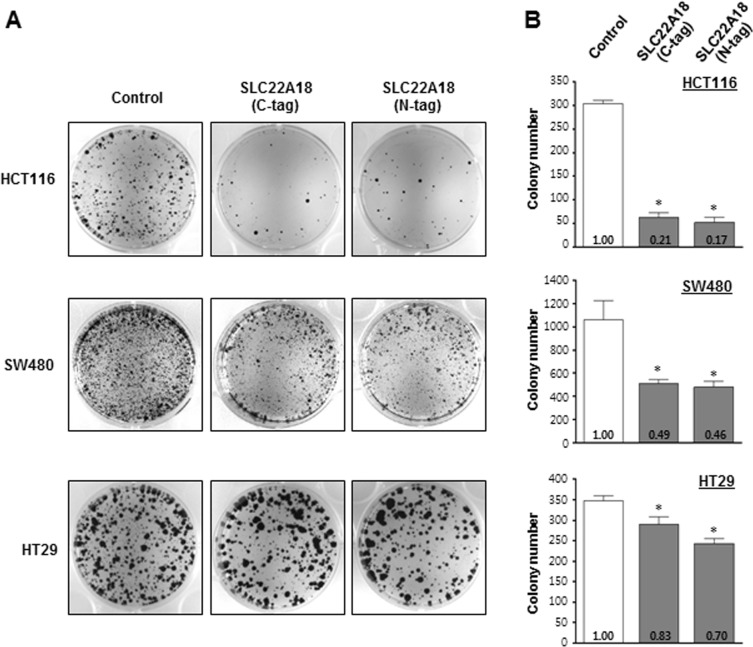
Examination of the tumor suppressor activity of SLC22A18 in colony formation assay **A.** Ectopic expression of SLC22A18 suppresses growth of CRC cell lines. Two *SLC22A18*-expression vectors with V5 or Xpress epitopes respectively at the C- or N-termini have been prepared and transfected to three independent CRC cells, HCT116, SW480, and HT29. For the control, pcDNA3.1 plasmid with no insert has been used. Colonies after G418 selection and Coomassie staining are shown. Each transfection has been carried out three independent times. **B.** Bar graphs showing results from colony formation assay. For all three cell lines, transfection of *SLC22A18* expression plasmids led to statistically significant decrease in colony numbers compared to control plasmid transfection. (*) represents *P*-value of < 0.05. Note the particularly strong effect in the case of HCT116 cells.

We next examined if SLC22A18 has tumor suppressor activity using colony formation assay. Three CRC cell lines, HCT116, SW480, and HT29 were selected, and two expression constructs of the full length SLC22A18 with epitopes on the N-terminus or C-terminus were generated. Both constructs showed similar effects in inhibiting formation of colonies, and all three cell lines tested showed inhibited colony formation (Figure 2A) upon ectopic expression of SLC22A18 although the inhibitory effect of SLC22A18 differed considerably among the three cell lines (Figure 2B). HCT116 showed the most dramatic response in that the number of colonies was only about 20% of what was seen in the control transfection case. Subsequent experiments were mostly carried out with HCT116 cells.

### G2/M cell cycle arrest induction by SLC22A18

**Figure 3:**
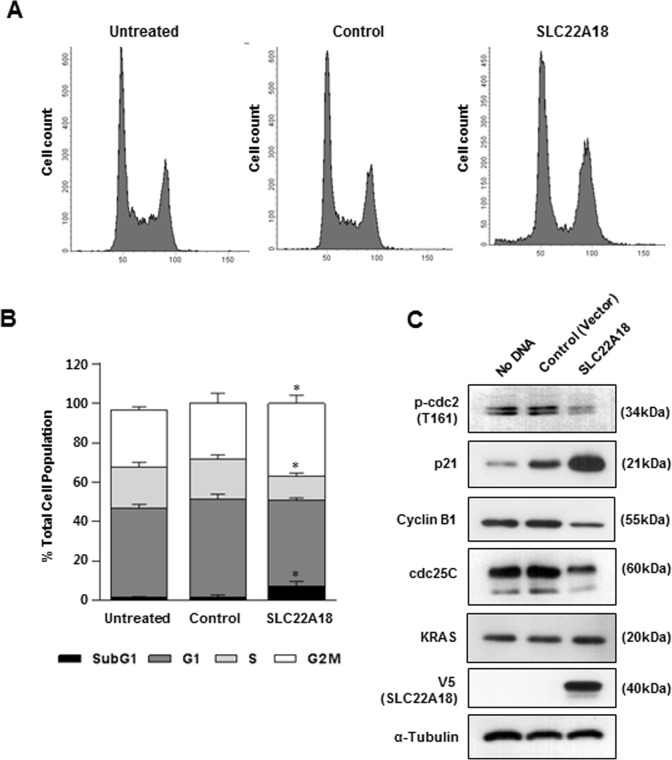
G2/M arrest induction by SLC22A18 **A.** Cells were infected with control virus or V5-tagged SLC22A18 virus and cultured for 48 hours. Uninfected cells were used as an additional control. Cells were collected and PI-stained after 48 hours, and the DNA contents were measured by flow cytometry. A representative result is shown. **B.** Graphic representation of the proportion of cells in each cell cycle phase from flow cytometric analyses. Data were obtained from three independent trials. Decrease and increase respectively in the proportions of cells in S-phase and G2/M phase were seen. (*) represents *P*-value of < 0.05 compared to control vector condition. **C.** Immunoblot analysis of HCT116 cell lysates showed that SLC22A18 induced changes in the level of cell cycle markers consistent with G2/M arrest. Note the up-regulation of p21 and down regulation of phospho-cdc2, cyclin B1 and cdc25C. V5-positive band is shown only in *SLC22A18*-virus infected cells. KRAS and α-tubulin show no change.

Tumor suppressors often induce cell cycle arrest. We therefore examined the effect of ectopic SLC22A18 expression on cell cycle progression in HCT116 cells using PI staining in combination with flow cytometric analyses. V5 epitope-tagged *SLC22A18* was incorporated into a retroviral vector with IRES-GFP in tandem. Control virus expressed only GFP. Compared to cells not infected or infected with the control virus, SLC22A18 virus-infected cells showed a significant decrease in the fraction of cells in S phase and increase in the fraction in G2 phase consistent with induction of G2/M arrest (Figure 3A, 3B). The sub-G1 fraction also showed a low level albeit statistically significant increase (Figure 3B). The extent of the increase suggests that apoptosis which typically follows prolonged G2/M arrest was taking place. We also checked for changes in cell cycle markers after ectopic expression of SLC22A18. Again, consistent with G2/M arrest, we saw decreased levels of p-Cdc2, Cyclin B1 and Cdc25c and an increase in the level of p21 in cells transduced with SLC22A18 virus compared to control cells (Figure 3C). Taken together with inhibition of colony formation, the G2/M arrest induced by SLC22A18 strongly suggests that SLC22A18 indeed functions as a tumor suppressor.

### Negative interaction between SLC22A18 and KRAS

**Figure 4:**
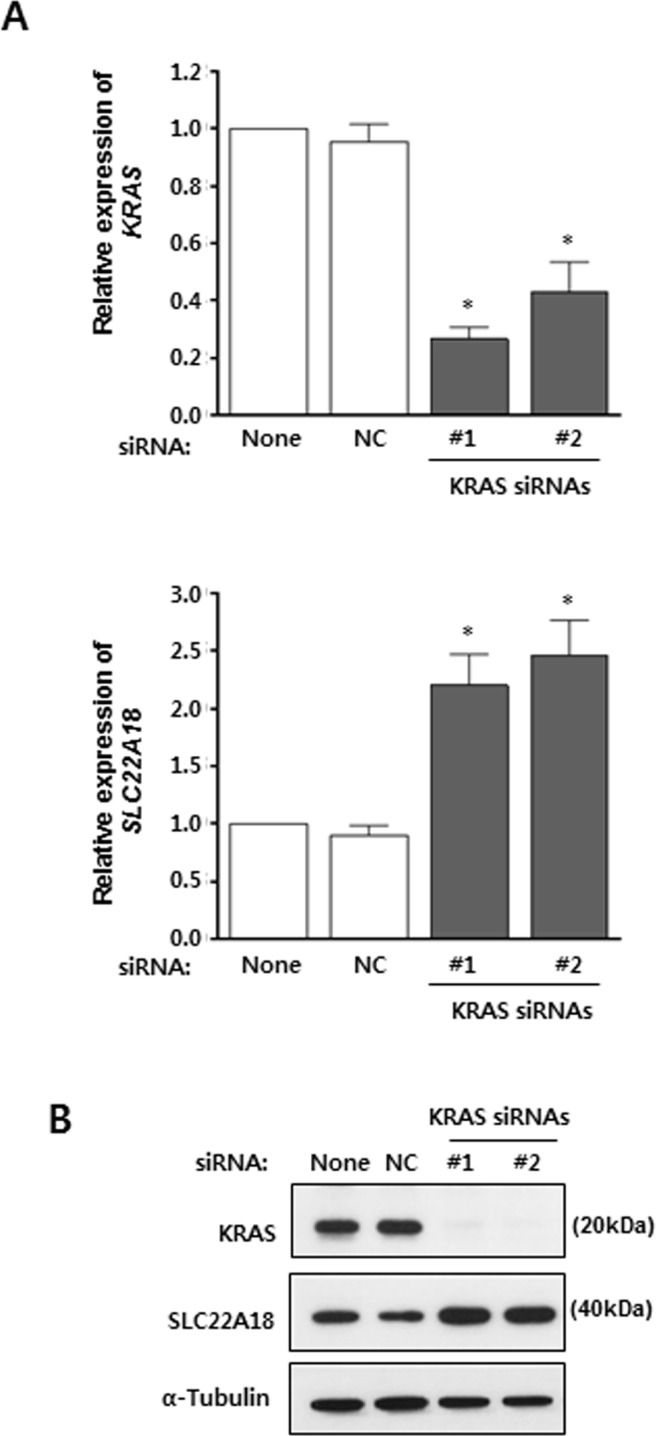
KRAS suppresses *SLC22A18* expression **A.** Real-time RT-PCR analyses using HCT116 cells transfected with siRNAs. The *KRAS* level is expressed relative to the level seen in transfection reagent only control. Knock-down of *KRAS* mRNA by two specific siRNAs is shown (top panel). NC represents non-targeting control siRNA transfection. Note that *SLC22A18* level increased over 2 fold (bottom panel). The graphs show results as mean ± S.D. of three independent experiments. (*) represents *P*-value of < 0.05 compared to NC. **B.** Immunoblot analyses of siRNA transfection assay. HCT116 lysates were examined after transfection of siRNAs targeting *KRAS* mRNA. Treatment with the transfection reagent only and transfection of control siRNA were used as controls. Knock-down of KRAS and elevation of SLC22A18 proteins were seen. For the loading control, α-tubulin was used.

Oncogenic mutations in *KRAS* gene is found in up to 50% of CRC cases [18]. Specifically, mutations of Gly12 or Gly13 lead to constitutive activation of KRAS signaling pathway. HCT116 contains the G13D mutation of *KRAS* which allows a chance to see if KRAS signaling affects expression of *SLC22A18*. First, two independent siRNAs targeting *KRAS* mRNA were shown to induce efficient down-regulation of *KRAS* by real-time RT-PCR (Figure 4A). Their effects were also confirmed by immunoblotting (Figure 4B). Importantly, *SLC22A18* expression in HCT116 cells was shown to be up-regulated upon specific targeting of *KRAS* mRNA (Figure 4A). Again, this was confirmed by immunoblotting for SLC22A18 (Figure 4B). These observations are consistent with that KRAS signaling down-regulates SLC22A18 as part of its pro-proliferation activity.

**Figure 5:**
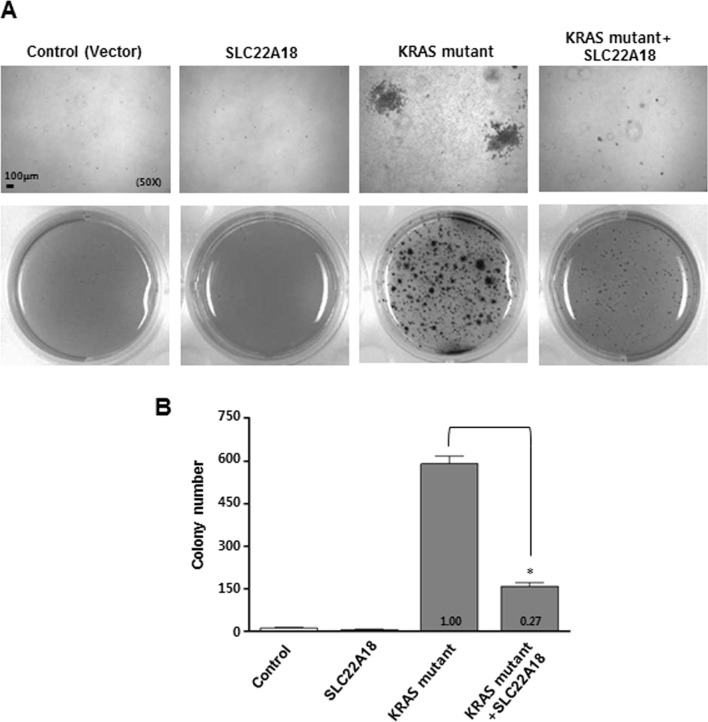
SLC22A18 inhibits KRAS-induced anchorage independent growth of NIH3T3 cells Cells were infected with indicated viruses or combination of viruses. Cells were allowed to grow on soft agar for 21 days and then stained and counted. Representative images are shown. The number and size of anchorage independent colonies seen with the infection of *KRAS*-encoding virus both decreased when super-infected with *SLC22A18*-encoding virus. Upper panels are higher magnification views. **B.** Results from soft agar assay are quantitated. Bars represent the average number of colonies, and error bars represent S.D. of three independent experiments. (*) represents *P*-value of < 0.05.

Anchorage independent growth is one of the hallmarks of cellular transformation. We used a well-established protocol for *KRAS*-induced anchorage independent growth of NIH3T3 cells on soft agar to test if SLC22A18 negatively affects KRAS signaling and consequently the transformation. Pseudotyped retroviruses expressing SLC22A18 or KRAS^G12D^ were generated and used to infect NIH3T3 cells which were subsequently grown in soft agar. Cells infected with either control or SLC22A18 virus showed virtually no growth in soft agar as expected (Figure 5A, 5B). In contrast, KRAS virus transduction led to a dramatic increase in the number of multicellular colonies consistent with induction of transformation. Interestingly, co-infection of SLC22A18 virus with KRAS virus led to a significant decrease in the number of visible multicellular colonies (Figure 5A, 5B). Taken together with KRAS-induced down-regulation of *SLC22A18*, this result indicates that SLC22A18 and KRAS form a mutually inhibitory regulatory loop.

### Inhibition of xenograft tumor growth by SLC22A18

**Figure 6:**
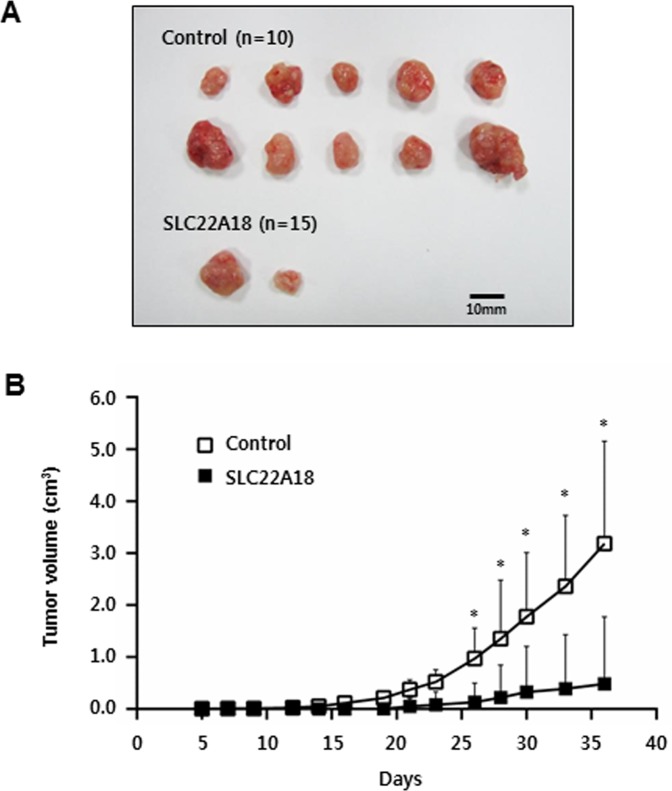
SLC22A18 suppresses growth of HCT116 xenograft tumors **A.** All tumors isolated from mice are shown. Note all 10 mice inoculated with control cells showed visible tumor growth while only 2 out of 15 mice inoculated with SLC22A18 expressing cells did so. **B.** Growth curves of xenograft tumors from the experiments with BALB/c nude mice. Changes in tumor volumes measured at the indicated days are shown. Error bars represent S.D. of tumor volumes, and (*) represents *P*-value of < 0.01.

We next examined the effect of SLC22A18 expression using a xenograft model. HCT116 cells infected either with control virus or with SLC22A18 virus were used to inoculate female BALB/c Slc-nu/nu mice. Tumor volumes were measured over the course of 5 weeks. A definitive and statistically significant difference was noted (Figure 6 and Supplementary Figure 4) between control group (*n* = 10) and SLC22A18 group (*n* = 15). Specifically, while all 10 control mice developed tumors, only 2 out of 15 from SLC22A18 did so, consistent with tumor suppressor activity of SLC22A18.

### Evaluation of *SLC22A18* as a prognostic marker of CRC

**Figure 7:**
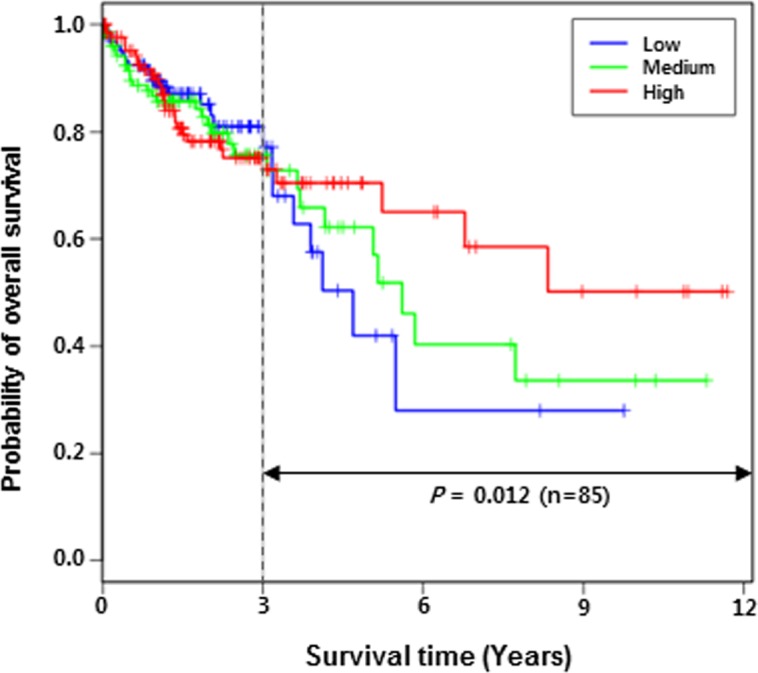
Lower expression of *SLC22A18* is correlated with lower long-term survival of COAD patients Patients were divided into three groups based on *SLC22A18* expression levels. Kaplan-Meier survival analysis shows that after three years, the survival rate is significantly co-related with the *SLC22A18* expression level (*P*-value estimated to be 0.012). Comparison between low and high groups led to a more significant correlation (*P*-value estimated to be 0.0032; see Results section).

*SLC22A18* has been previously reported as a potential prognostic marker in breast cancer and glioma [16, 17]. Thus, we investigated the relationship between *SLC22A18* expression level and patient survival using the TCGA COAD data. Patients were divided into three groups according to the expression levels of *SLC22A18* gene, and the resulting Kaplan-Meier survival curves are shown in Figure 7. Even though the survival analysis of 430 patients gave statistically insignificant result (*P*-value = 0.78), we observed that three curves were well separated beyond three years. Restricting the patient cohort to 85 survivors beyond three years, we obtained the *P*-value = 0.012. Comparison between two groups (55 patients in sum) of high and low expressions of *SLC22A18* yielded evidence for more significant difference (*P*-value = 0.0032). We have also carried out Cox Proportional Hazards regression analysis to determine the correlation between *SLC22A18* expression levels and survival based on individually matched data without categorizing patients into groups. We used TCGA survival data from 259 COAD patients whose gene expression data were uniformly obtained via HiSeq sequencing platform. The result supports conclusions from the grouping-based analysis. Specifically, even though the cohort of 259 patients gave overall a statistically insignificant result (*P*-value = 0.1939), restricting the patient cohorts to 84 survivors beyond two years and to 58 survivors beyond 3 years led to *P*-values of 0.0312 and 0.0190 respectively. This suggests that the expression level of *SLC22A18* has a predictive power for long-term survival in COAD patients.

## DISCUSSION

Multiple lines of evidence have suggested that SLC22A18 functions as a tumor suppressor. They include chromosomal location of the gene at 11p15.5 as well as missense mutations and aberrant splicing found in tumor tissues of various cancer types [7–9]. Furthermore, gene silencing and loss-of-heterozygosity of this paternally imprinted gene mostly occurs to the maternal allele [10–12]. Although low-level expression in glioma has been reported to be associated with poor prognosis [15], mechanistic studies for its tumor suppressor activity have been sparse. To the best of our knowledge, this study is first to report G2/M arrest induction by SLC22A18 and the mutually inhibitory activity with *KRAS*. Although inhibition of colony formation, induction of cell cycle arrest and inhibition of xenograft tumor growth were seen from overexpression of SLC22A18 which in itself could have toxic effects, epistatic relationship with KRAS supports that the observed tumor suppressor activities of SLC22A18 is endogenous to the gene.

Interestingly, the down-regulation of *SLC22A18* in tumor tissue is not seen in other types of cancer but is restricted to colon and rectal cancers although the analyses based on matched normal-tumor tissues were available only for a limited number of cancer types. Of note, at least in the case of glioma, for which TCGA does not provide matched sample data, one report indicated that *SLC22A18* functions as a tumor suppressor with lower expression in tumor tissues [15]. That *SLC22A18* functions as a tumor suppressor is strongly supported by down-regulated expression in CRC, but it remains to be determined if the protein can inhibit cell cycle progression or KRAS signaling in other types of cancer cells, particularly in those where mutations of *SLC22A18* have been found. We have also shown that *SLC22A18* is a potential prognostic marker for CRC: lower expression appears to be associated with poor long-term survival. Clearly however, the prognostic value of *SLC22A18* needs to be re-examined with a larger number of patients.

Among the critical remaining questions is how the organic ion transporting activity of SLC22A18 is connected with its tumor suppressor activity. The class of organic solutes which SLC22A18 transports is not known. In fact, the assignment of SLC22A18 as an organic ion transporter is based on the SLC22 subfamily membership but not on exact functional analysis. Still, it should be noted that SLC22A18 is expressed highly in organs with metabolite transport functions including intestine, kidney, placenta and liver [8]. Importantly, it has been proposed that solute carriers play key inter- and intra-cellular functions in sensing and signaling to maintain homeostasis in response to physiological changes [19]. Therefore, a better understanding of the exact function of SLC22A18 as a solute transporter is likely to be a step toward understanding its function as a tumor suppressor of CRC.

## MATERIALS AND METHODS

### Patient samples and RNA isolation from tissues

Primary colorectal cancer and noncancerous colon samples used in RT-PCR study have been previously described [5]. The study was approved by the local institutional review board. The procedures for tissue processing and RNA isolation have been described [5].

### Cell lines and culture conditions

Human colorectal carcinoma cells, HCT116, HT29 and SW480, were obtained from the American Type Culture Collection (ATCC, Manassas, VA). HCT116 and HT29 cells were cultured in McCoy's 5A, and SW480 cells were cultured in RPMI-1640 supplemented with 10% fetal bovine serum (Hyclone, Logan, UT). NIH3T3 fibroblast cells were also purchased from the ATCC and cultured in DMEM supplemented with 10% calf serum (Invitrogen, Carlsbad, CA).

### Real-time RT-PCR and conventional RT-PCR analysis

Total RNA from cultured cells was extracted using the TRI Reagent^®^ (Ambion, Austin, TX). Single-stranded cDNA was synthesized from 2 μg of total RNA using ImProm-II^™^ reverse transcriptase (Promega, Madison, WI). For the quantitative analysis of *SLC22A18* and *KRAS* mRNA levels, cDNA generated from 10 ng of total RNA was subjected to PCR amplification using Kapa SYBR Fast qPCR kit (Kapa Biosystems, Boston, MA) on a CFX96 Real-time PCR detection system (Bio-Rad, Hercules, CA). *ACTB* and *HPRT1*, two internal control genes, were used as dual reference genes. Primer sequences are available online (Supplementary Table 1). Cycling conditions were as follows: pre-denaturation for 2 min at 95°C, a 2-step reaction (40 cycles) of 10 sec at 95°C and 40 sec at 60°C, and a dissociation peak analysis. The mRNA expression value of target genes was calculated with Bio-Rad CFX Manager Software. For conventional RT-PCR analysis, cDNA was amplified using Platinum Taq DNA polymerase (Invitrogen) and *SLC22A18* primers. Cycling conditions were as follows: pre-denaturation for 2 min at 95°C, a 3-step reaction (28 cycles) of 15 sec at 94°C, 15 sec at 60°C and 30 sec at 72°C with a final extension of 10 min at 72°C. *ACTB* expression was used as an internal control, and PCR products were visualized by agarose gel electrophoresis.

### Immunohistochemical staining

Immunohistochemistry (IHC) for SLC22A18 was performed on 4-μm frozen sections of clinically obtained colon cancer tissues. Tissue sections were deparaffinized with xylene, hydrated in serial dilutions of alcohol, and immersed in 3% H_2_O_2_. Following antigen retrieval in citrate buffer (pH 6.0), the tissue sections were incubated with protein blocking agent (ScyTek, Logan, UT) to avoid non-specific antibody binding for 30 minutes at room temperature and then incubated overnight with primary antibody against SLC22A18 (1:400; LSBio, Seattle, WA) at 4°C in a humidified chamber. After washing with PBS three times, the sections were incubated with a biotinylated secondary antibody and streptavidin conjugated to horseradish peroxidase (ScyTek) for 60 minutes at room temperature followed by a PBS wash. The liquid 3, 3′-diaminobenzidine (ScyTek) for chromogen was used for development and was followed by counterstaining with Meyer's hematoxylin.

### Retrovirus vector construction

To generate retroviruses expressing SLC22A18 or KRAS^G12D^, the coding region of each gene was subcloned into the LZRS retroviral vector plasmid (pLZRS) along with IRES-GFP (internal ribosome entry site-green fluorescent protein) as previously described [20, 21]. The control virus contained just the IRES-GFP in pLZRS. Production of high titer virus preparation in 293GPG cells was carried out as described with minor modifications [22]. Further details on construction of viral vectors and generation of pseudotyped viral particles will be provided upon request. Retroviruses encoding *SLC22A18* or *KRAS* were infected with polybrene (4 μg/ml) into HCT116 or NIH3T3 cells. After 48 hours, the efficiency of infection was determined using fluorescence from GFP.

### Flow cytometry

Two days after retroviral infection, the cells were trypsinized and rinsed with ice-cold PBS and resuspended at a concentration of 7×10^5^ cells/ml in PBS. After fixation with 70% ethanol and washing with PBS, the cells were stained with 50 μg/ml propidium iodide (Sigma, St. Louis, MO) in PBS containing 0.1% Triton X-100 and 1 μg/ml RNase (Sigma) for 20 min. Flow cytometric analysis was carried out on a BD LSRFortessa cell analyzer (BD Biosciences, San Jose, CA). Typically, fluorescence distribution of a total of 10,000 nuclei was analyzed using BD FACSDiva™ software.

### Transfection of siRNAs

Specific siRNAs for *KRAS* and the scrambled negative control (NC) siRNA were purchased from Qiagen (Germany). Target sequences are available on line (Supplementary Table 2). The siRNA duplexes were transfected to 1.5×10^5^ cells in 35mm dishes using Lipofectamine RNAiMAX (Invitrogen) at the concentration of 50 nM for 48 hours according to the manufacturer's instructions.

### Western blotting analysis

For immunoblotting, cells were lysed in cold lysis buffer (50 mM Tris–Cl, pH 8.0, 150 mM NaCl, 2 mM Na_2_EDTA, 1% NP-40, 0.1% sodium dodecyl sulfate, 0.5% Na-deoxycholate, and 10 mM NaF) supplemented with a mixture of protease inhibitors (Sigma) and phosphatase inhibitors (Sigma). After incubating on ice for 20 min, the supernatants were isolated by centrifugation at 13,000 rpm for 20 min. The concentration of total protein was determined using the BCA protein assay kit (Thermo Scientific Pierce, Rockford, IL). For detection of SLC22A18, cells were resuspended in Laemmli buffer, boiled for 10 min and centrifuged. Antibodies against p-cdc2 (T161), p21 Waf1/Cip1 (12D1), Cyclin B1 and cdc25C were purchased from Cell Signaling Technology (Beverly, MA). Monoclonal antibody against the V5 epitope was purchased from Invitrogen. Antibodies to KRAS, SLC22A18 and α-Tubulin were purchased from Santa Cruz Biotechnology (Santa Cruz, CA), LSbio and AbFrontier (Seoul, Korea), respectively. Proteins were detected using peroxidase-conjugated anti-mouse-IgG or anti-rabbit-IgG antibodies in combination with enhanced chemiluminescence detection kit (Amersham-Pharmacia Biotech, Piscataway, NJ).

### Colony formation assay

Two *SLC22A18* expression plasmids, one with V5 epitope in the C-terminus and another with Xpress epitope in the N-terminus were constructed in pcDNA3.1 vector. Cells (1.5×10^5^) were seeded in a 35mm dish and transfected with *SLC22A18* plasmids or pcDNA3.1 plasmid (2 μg each) using Lipofectamine 2000 (Invitrogen) for HCT116 cells and HT29 and Lipofectamine LTX (Invitrogen) for SW480 cells following the manufacturer's protocols. Forty-eight hours after transfection, HCT116 and HT29 cells were placed under selection with G418 at 800 μg/ml (Invitrogen) respectively for 10 and 18 days. In the case of SW480, cells were treated with G418 at 800 μg/ml for 8 days initially and at 400 μg/ml for 6 days subsequently. Colonies were stained with 0.1% Coomassie Blue in 45% methanol and 10% acetic acids solution, and the colony numbers were determined using Gel Doc XR system (Bio-Rad) with Quantity One^®^ 1-D analysis software (Bio-Rad).

### Soft agar assay

For soft agar assays, 5×10^3^ (per 21mm dish) retrovirus-transduced NIH3T3 cells were suspended in 0.35% agar containing DMEM with 10% calf serum and overlaid onto a 0.9% agar solution. Colonies were stained after 21 days with MTT solution at the concentration of 1 mg/ml overnight. Colony numbers were determined using Gel Doc XR system with Quantity One^®^ 1-D analysis software. Details are available upon request.

### Tumor xenograft experiments

One day after infection with control viruses or viruses encoding *SLC22A18*, HCT116 cells were trypsinized and counted. A total of 2 × 10^6^ cells in 100 μL of phosphate-buffered saline were injected subcutaneously into the right flank of 7-week-old female BALB/c Slc-nu/nu mice (Japan SLC, Japan). Tumor size was measured three times weekly with calipers for approximately 5 weeks, and the tumor volume was calculated using the equation: volume = (W^2^xL)/2 (W, the shortest dimension; L, the longest dimension). This study was reviewed and approved by the Institutional Animal Care and Use Committee (IACUC) of Ewha Womans University.

### Statistical analysis

Paired *t*-test (one- or two-tailed) was used to evaluate the significance of results from expression analyses and colony formation, cell cycle and soft agar assays. For tumor xenograft experiments, unpaired *t*-test was used.

### Analysis of the TCGA COAD data

Gene expression data from TCGA colon adenocarcinoma (COAD) consortium were downloaded at level 3 from the Broad GDAC Firehose website (released on April 16, 2014). RNA-Seq experiments of TCGA COAD had been performed in two different Illumina platforms of HiSeq and Genome Analyzer for 430 patients (*n* = 259 and 171, respectively), and *SLC22A18* expression was analyzed separately from each platform data. For survival analysis, patients were divided into three groups according to *SLC22A18* expression levels. The log-rank test was performed for Kaplan-Meier survival analysis of three groups using the Survival package version 2.37-7 (http://CRAN.R-project.org/package=survival).

## SUPPLEMENTARY MATERIAL FIGURES AND TABLES


