# Supplementary material for: Characterization of SLC22A18 as a tumor suppressor and novel biomarker in colorectal cancer
Source: Oncotarget. 2015 Jun 28;6(28):25368–80. doi: 10.18632/oncotarget.4681 (PMC4694837; doi:10.18632/oncotarget.4681)
Supplement: Supplementary file 1 [file oncotarget-06-25368-s001.pdf]

Characterization of *SLC22A18* as a tumor suppressor and novel biomarker in colorectal cancer

Supplementary Material

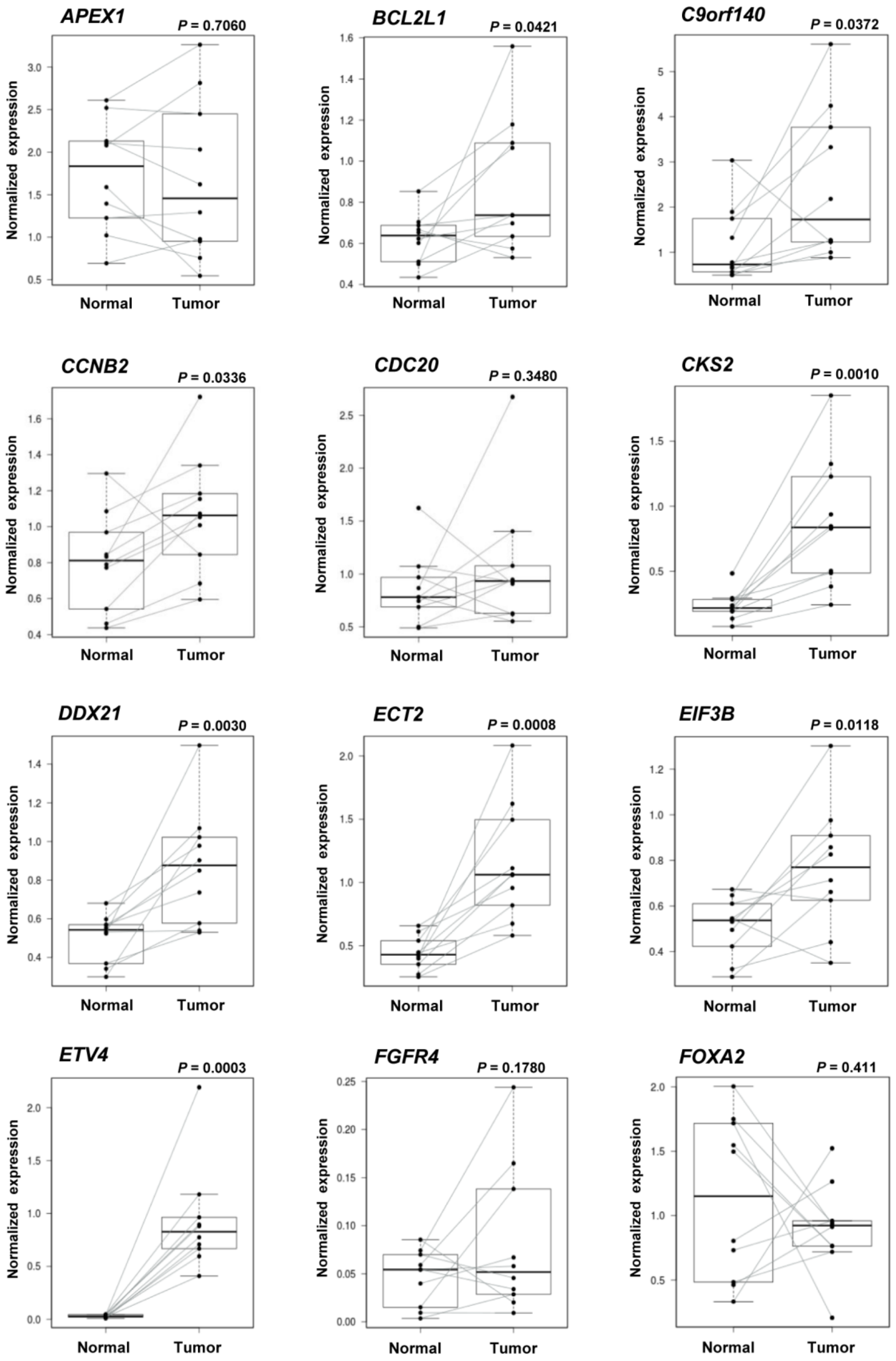

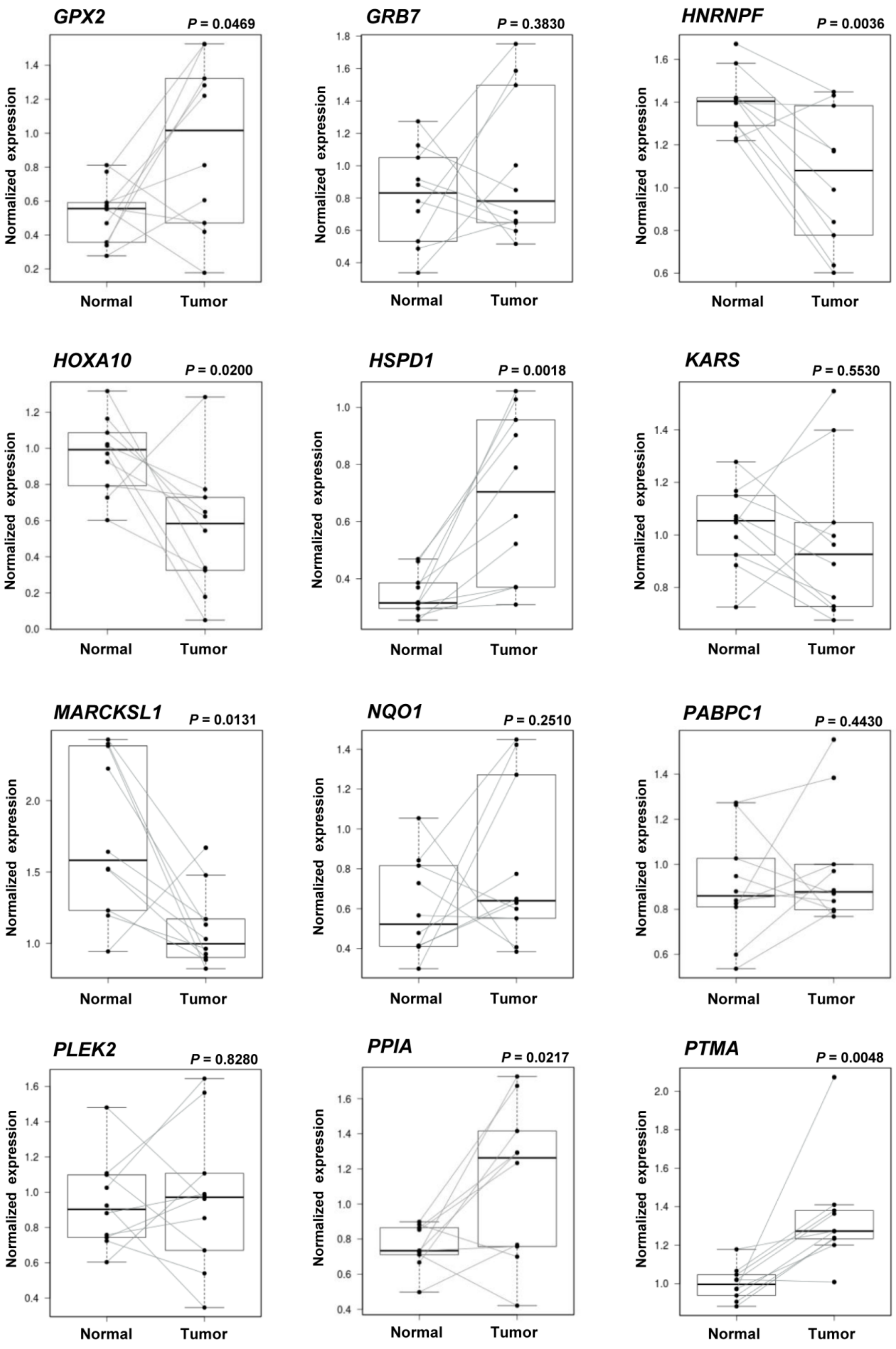

GPX2
P = 0.0469
GRB7
P = 0.3830
HNRNPF
P = 0.0036
HOXA10
P = 0.0200
HSPD1
P = 0.0018
KARS
P = 0.5530
MARCKSL1
P = 0.0131
NQO1
P = 0.2510
PABPC1
P = 0.4430
PLEK2
P = 0.8280
PPIA
P = 0.0217
PTMA
P = 0.0048
Normalized expression
Normal
Tumor

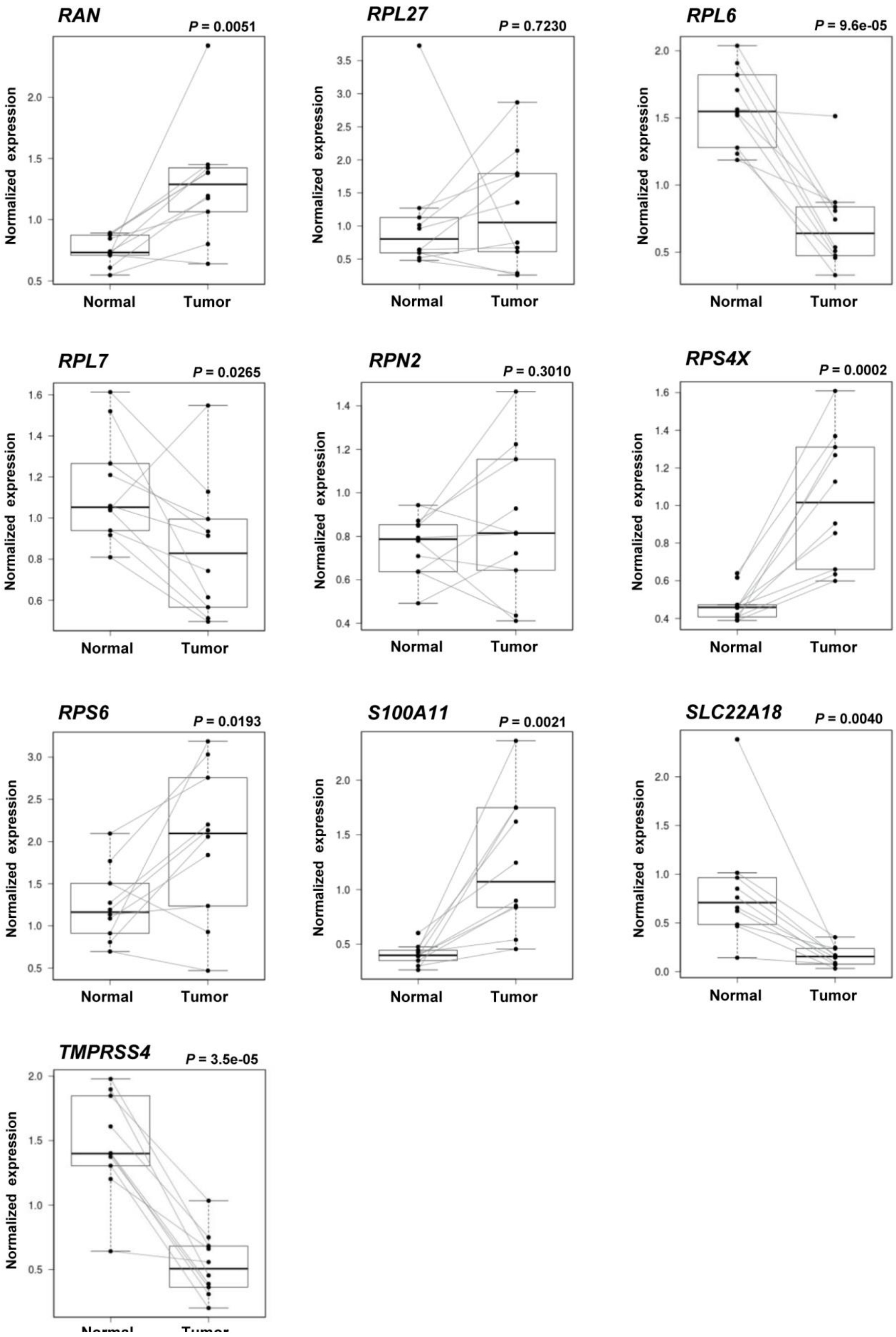
RAN
P = 0.0051
RPL27
P = 0.7230
RPL6
P = 9.6e-05
RPL7
P = 0.0265
RPN2
P = 0.3010
RPS4X
P = 0.0002
RPS6
P = 0.0193
S100A11
P = 0.0021
SLC22A18
P = 0.0040
TMPRSS4
P = 3.5e-05
Normalized expression
Normal
Tumor

Primer Sequences used Real-time PCR for 34 colon cancer biomarker genes

Gene	NCBI No of mRNA Reference Sequence	Primer sequence (Forward, 5'->3')	Primer sequence (Reverse, 5'->3')	Amplicon size (bp)
APEX1	NM_001641	gcttcgagcctggattaaga	gaccagtattgatgagagagtcca	147
BCL2L1	NM_001191	gctgagttaccggcatcc	agattctgaagggagagaaagaga	127
C9orf140	NM_178448	acgtcggaggcacaccat	cgccatcatctccaaacc	109
CCNB2	NM_004701	tgtacatgtgcgttggcatt	gaagccaagagcacagcagt	100
CDC20	NM_001255	cattcgcatctggaatgtgt	gcctgagatgagctccttgta	109
CKS2	NM_001827	ttcgacgaacactacgagtacc	agcctagactctgttggacacc	124
DDX21	NM_004728	ccagcaaagatgccatcag	gtgctgctgccagagctt	120
ECT2	NM_018098	gttctggggaagcattcaaa	aagcattgacactgatttcttgag	100
EIF3B	NM_001037283	acgacgtgagcgaggaag	tgtccactacaatcaccgaatc	90
ETV4	NM_001079675	ggacttcgcctacgactcag	cgcagaggtttctcatagcc	120
FGFR4	NM_002011	gcatccgctataactacctgcta	tacaccttgcacagcagctc	127
FOXA2	NM_021784	gagcggtgaagatggaagg	gtacgtgttcatgccgttca	122
GPX2	NM_002083	gcaaccaatttggacatcag	gcccattcacctcacatt	126
GRB7	NM_001030002	cttccgcctcttcaagtacg	ggccaccagggtattatctg	127
HNRNPF	NM_001098204	gttgagcttcggtggaattt	atgccttcagtgggagacac	128
HOXA10	NM_018951	ttccgagagcagcaaagc	cagtgtctggtgcttcgtgt	130
HSPD1	NM_002156	gcaaagttcctcagaagttggt	gcagcatccaataaagcagtt	114
KARS	NM_005548	acatctcactcactgacttcatcc	cagaagctcttttggcatgg	111
MARCKSL1	NM_023009	catcatgggcagccagag	cttttcacgtggccattctc	111
NQO1	NM_000903	cagctcaccgagagcctagt	gagtgagccagtacgatcagtg	123
PABPC1	NM_002568	ctatggatttgtacactttgagacg	cgagacttaaatcgtccaacaa	111
PLEK2	NM_016445	atggctgcaccatcacct	agaacaggcctccaggaagt	104
PPIA	NM_021130	cctaaagcatacgggtcctg	tttcactttgccaaacacca	133
PTMA	NM_001099285	cagctttatcgccagagtcc	agtccttggtggtgatttcg	124
RAN	NM_006325	ggcgcttctggaaggaac	acgtttcacgaaggtcgttt	111
RPL27	NM_000988	gacgcaaagctgtcatcgt	cttggcgatcttcttcttgc	134
RPL6	NM_000970	ccaggaaggtgagatcttcg	gccctggagctgaggaatag	127
RPL7	NM_000971	ccaaaagatgcttcgaaagg	cagcttttcttgccatcctc	122
RPN2	NM_002951	agacagccaccagaacttcg	tctggttatggagtcggaca	101
RPS4X	NM_001007	acggaggtcctctttccttg	ccggtcaatttatccagcat	106
RPS6	NM_001010	aggcgttcagctgcttca	tacgaagtttgcgttcatcg	96
S100A11	NM_005620	tgaggagaggctccagacc	accgctcagtctctgtaggg	127
SLC22A18	NM_002555	tacctgcaaaccaccttcg	agcaggtagagcgccaag	128
TMPRSS4	NM_001083947	tcatcattgtggttgtcctca	agtccagctctccgtcacac	111

Supplementary Figure 1: Real-time RT-PCR analyses of candidate markers of CRC using normal-tumor matched patient samples. The meta-analysis pipeline for isolation of 34 candidate biomarker genes have been described previously [5]. Out of our patient samples, 10 were randomly selected for real-time RT-PCR analysis. Two-tailed *t*-test was applied for evaluation of the statistical significance. Some of the genes elevated in tumor tissues has been characterized as potential biomarkers of CRC and for functional interaction with cMYC and KRAS [5]. Four genes, *HNRNPF*, *RPL6*, *SLC22A18* and *TMPRSS4* show statistically significant decrease in tumor tissues (*P*-value of <0.01). Primers used for each of the 34 genes are presented in the table.

Patient S1

H&E (X1)

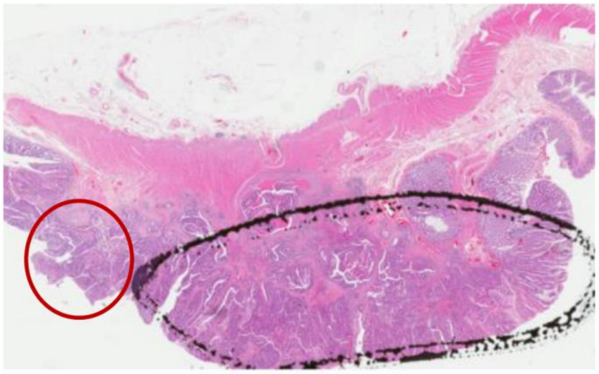

Anti-SLC22A18 (X1)

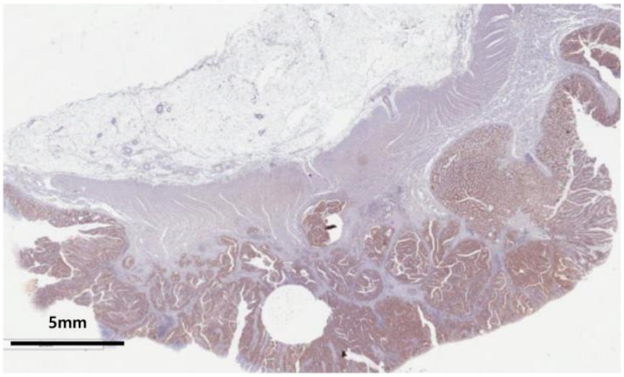

Anti-SLC22A18 (X200)_N, T

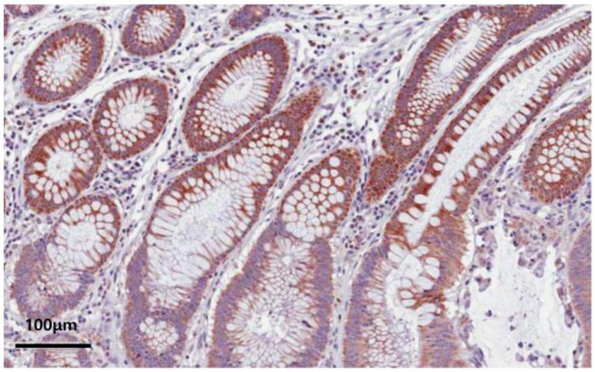

Patient S2

H&E (X1)

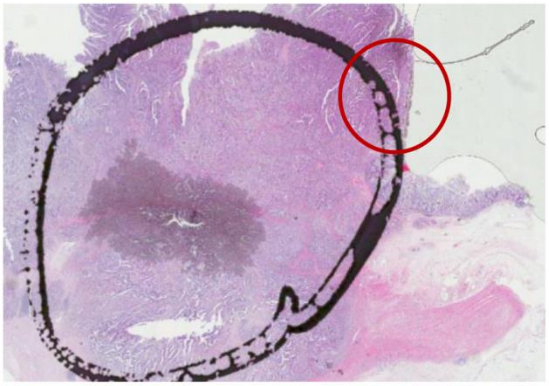

Anti-SLC22A18 (X1)

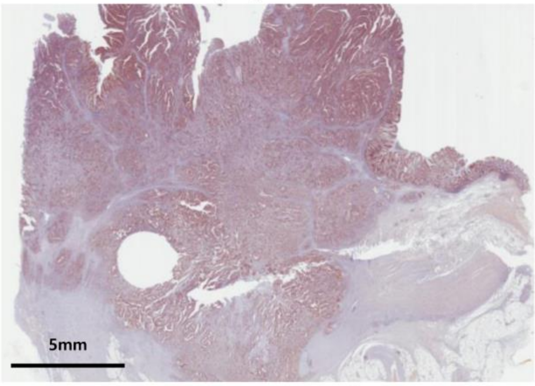

Anti-SLC22A18 (X100)_N, T

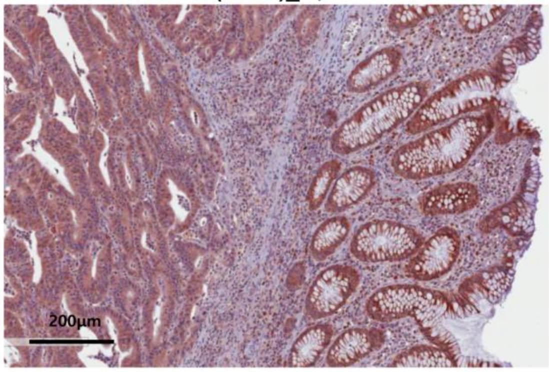

Anti-SLC22A18 (X200)_N, T

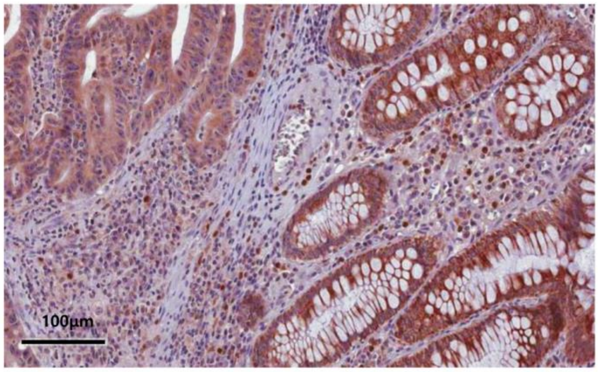

Patient S3

H&E (X1)

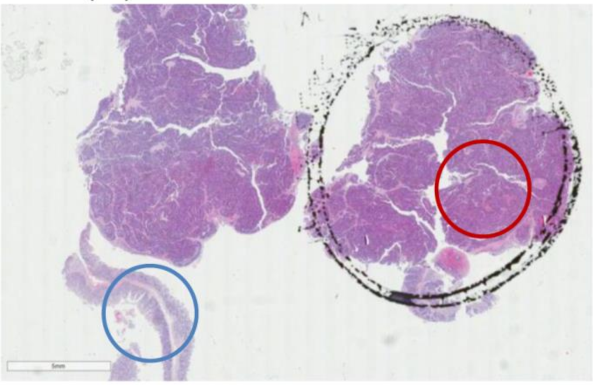

Anti-SLC22A18 (X1)

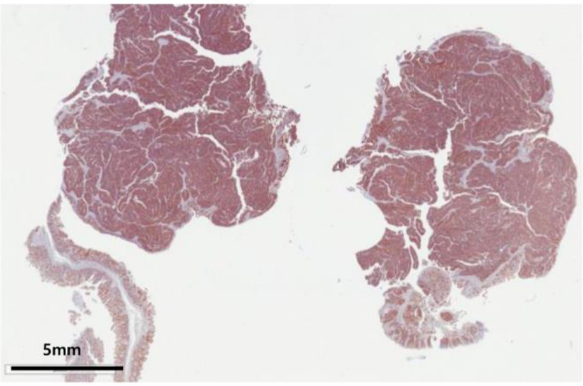

Anti-SLC22A18 (X200)_N, T

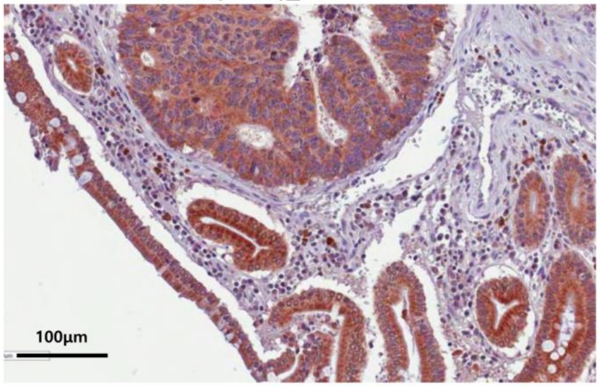

Anti-SLC22A18 (X200)_T

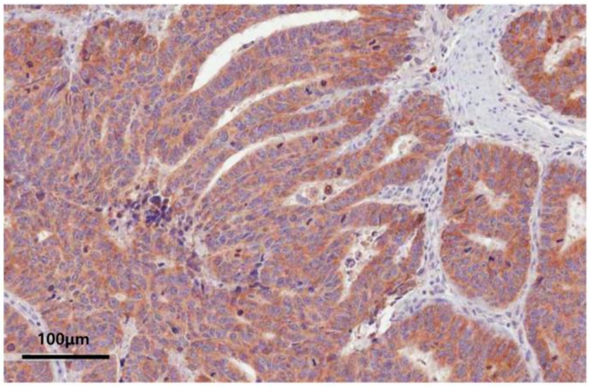

Patient S4

H&E (X1)

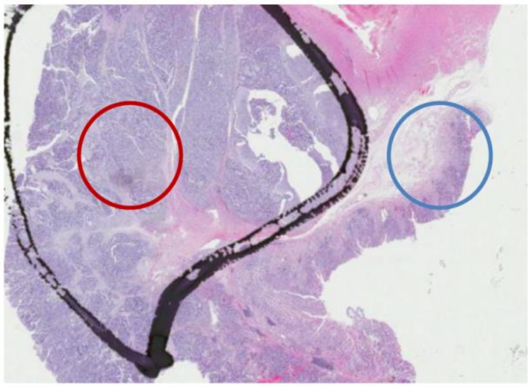

Anti-SLC22A18 (X1)

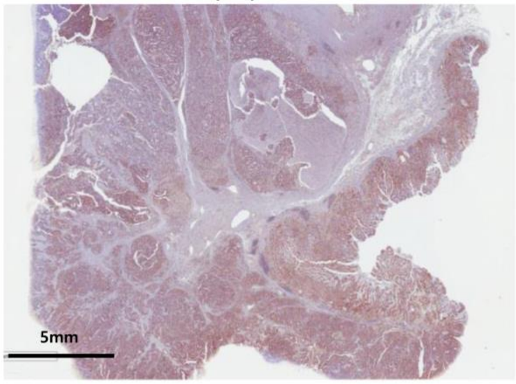

Anti-SLC22A18 (X200)_N

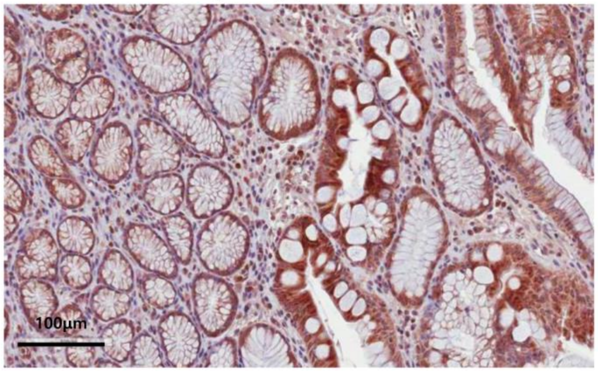

Anti-SLC22A18 (X200)_T

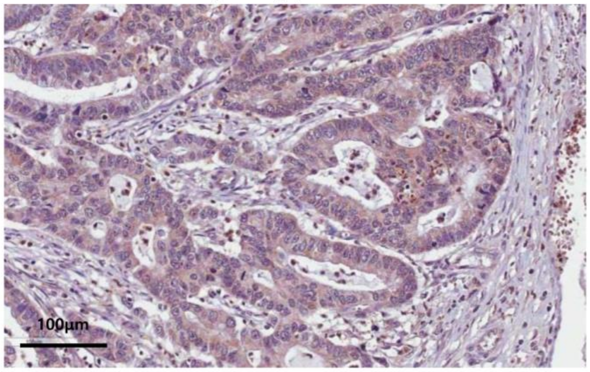

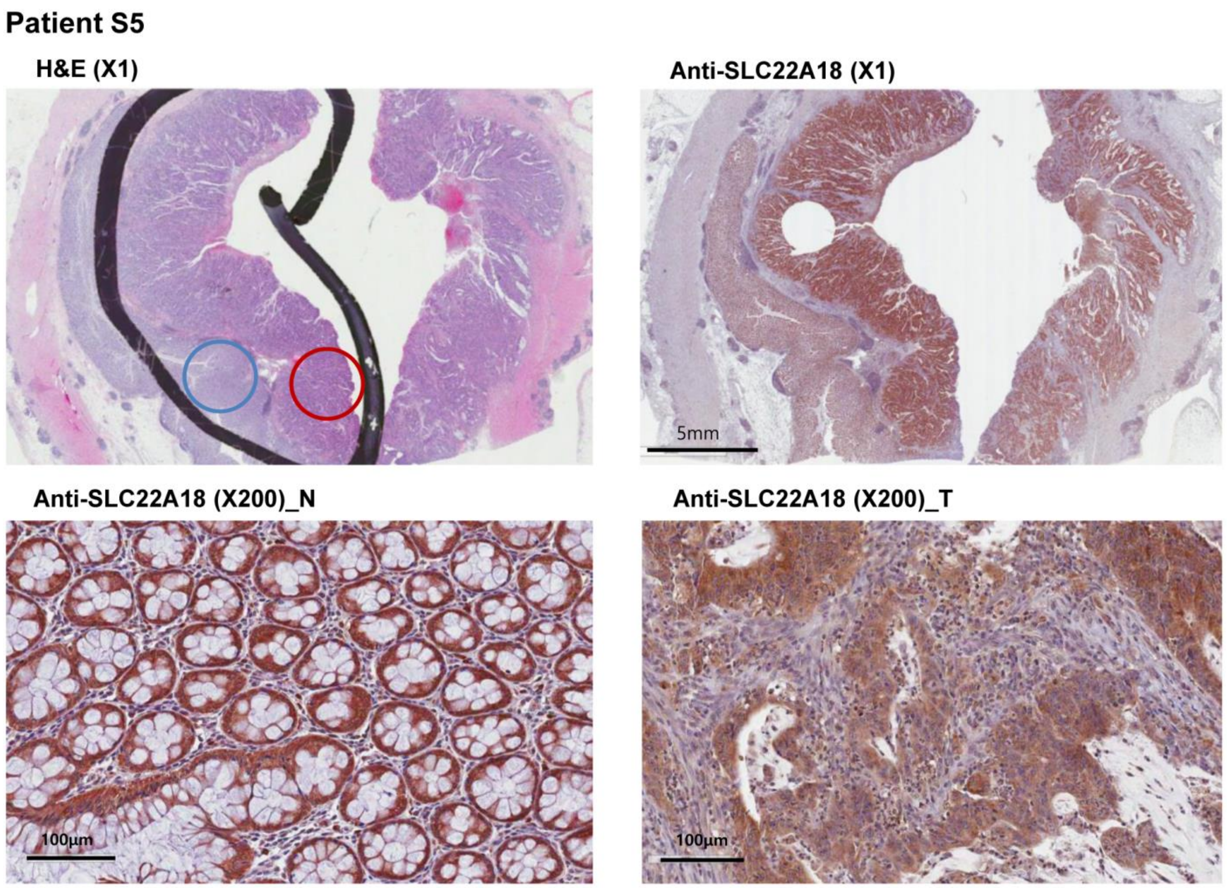

Supplementary Figure 2: Immunohistochemical staining of CRC tissue samples from 5 independent patients. Black markings on H&E slides indicate tumor masses. Next to H&E slides, immunohistochemical staining with anti-SLC22A18 antibody is shown. For patients S1 and S2, an area containing both normal (N) and tumor (T) tissues was selected (red circle) for enlarged views presented below (enlargement folds are indicated as x1, x100 and x200). Note the x100 view of patient S1 is presented as Figure 1D. For the other patients, separate areas were chosen for enlarged views. Blue circles contain normal tissues and red circles contain tumor tissues.

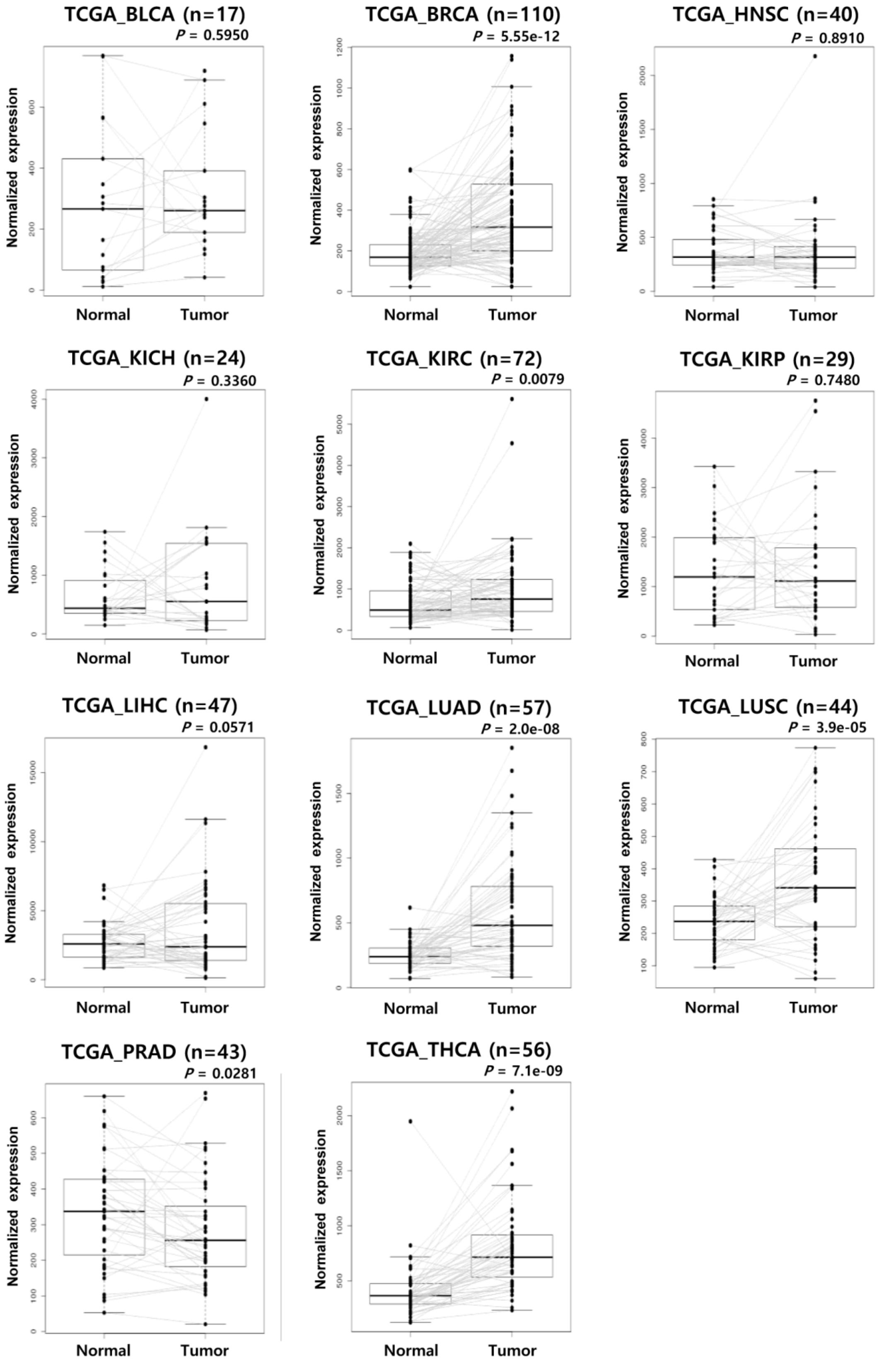

BLCA, Bladder Urothelial Carcinoma; BRCA, Breast invasive carcinoma; HNSC, Head and Neck squamous cell carcinoma; KICH, Kidney Chromophobe; KIRC, Kidney renal clear cell carcinoma; KIRP, Kidney renal papillary cell carcinoma; LIHC, Liver hepatocellular carcinoma; LUAD, Lung adenocarcinoma; LUSC, Lung squamous cell carcinoma; PRAD, Prostate adenocarcinoma; THCA, Thyroid carcinoma

Supplementary Figure 3: Expression analyses of *SLC22A18* using matched normal-tumor patient data from TCGA. The gene expression data (illumina hiseq RNAseq v2 gene expression (RSEM normalized)) were downloaded via Broad GDAC Fire website (released on April 16, 2014). In order to confirm down-regulation of *SLC22A18*, we examined only 12 of 25 cancer types including COAD with over 10 matched normal-tumor samples. Two-tailed *t*-test was applied to analyze differences in *SLC22A18* expression between matched normal and tumor samples. Note only COAD (Figure 1C) show consistent down-regulation in tumor tissues.

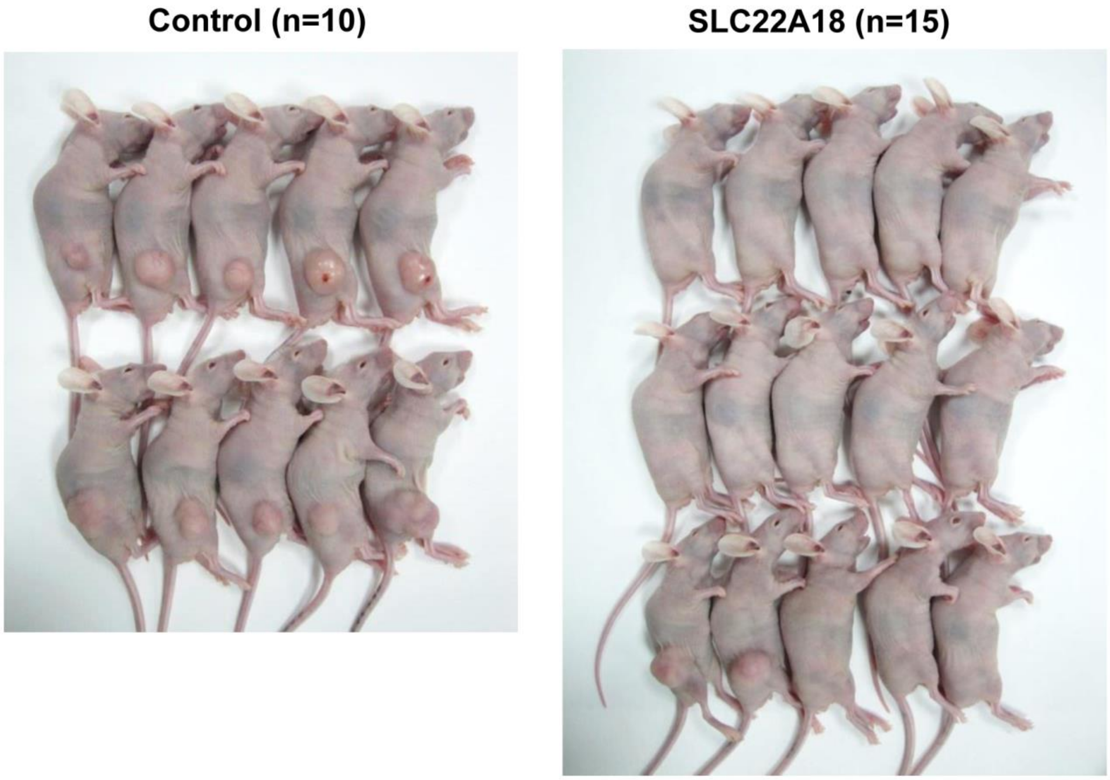

Supplementary Figure 4: HCT116 xenograft tumor experiments using athymic nude mice. Mice were divided into two groups and inoculated with $2x10^6$ cells infected either with control viruses or with *SLC22A18*-expressing viruses. Images show mice sacrificed after 5 weeks of tumor growth. See Figure 6 for comparative measurements of tumor growth.

Supplementary Table 1

Sequences of oligonucleotide primers used in RT-PCR.

Gene	Primer sequence (Forward, 5'- to 3'-)	Primer sequence (Reverse 5'- to 3'-)	Amplicon size (bp)	Usage
SLC22A18	tacctgcaaaccaccttcg	agcaggtagagcgccaag	128	For real-time PCR
SLC22A18	ccaagctggctacctcatgt	caggaggcagaagtggaaga	181	For conventional PCR
KRAS	aggcctgctgaaaatgactg	tccctcattgcactgtactcc	219	For real-time and conventional PCR
ACTB	ccaaccgcgagaagatga	tccatcacgatgccagtg	121	For real-time and conventional PCR
HPRT1	tgaccttgatttattttgcatacc	cgagcaagacgttcagtcct	102	For real-time PCR

Supplementary Table 2

Sequences of *KRAS* siRNAs used in RNA interference.

Name	siRNA Sequence (Sense strand)
KRAS siRNA #1	5'-GTGGACGAATATGATCCAACA-3'
KRAS siRNA #2	5'-CAGACGTATATTGTATCATTT-3'
